# Latent class analysis of depression among patients with non-small cell lung cancer after surgery: A cross-sectional study

**DOI:** 10.1371/journal.pone.0324723

**Published:** 2025-06-17

**Authors:** Xiaoxu Wang, Jinxin Liu, Cuicui Li, Haiyang Duan, Hengxiao Lu, Qiaona Dong, Ruijuan Sun

**Affiliations:** 1 College of Nursing, Shandong Second Medical University, Weifang, Shandong Province, China; 2 Thoracic surgery medical center, The First Affiliated Hospital of Shandong Second Medical University (Weifang People’s Hospital), Weifang, Shandong Province, China; 3 Department of Critical Medicine, The First Affiliated Hospital of Shandong Second Medical University (Weifang People’s Hospital), Weifang, Shandong Province, China; Shandong Cancer Hospital and Institute Shandong First Medical University and Shandong Academy of Medical Sciences: Shandong Cancer Hospital and Institute, CHINA

## Abstract

**Background:**

Depression exhibits heterogeneity. However, limited research has explored this heterogeneity in patients with non-small cell lung cancer (NSCLC) after surgery from a person-centered perspective. This study aimed to identify classes of depression using latent class analysis (LCA) in patients with NSCLC after surgery and to explore the association between these classes and demographic and clinical characteristics, physical symptoms, distress disclosure, and relationship quality.

**Methods:**

A cross-sectional study was conducted with 234 patients with NSCLC in China from March 17, 2024 to May 16, 2024. Participants provided written informed consent before participating in the study. LCA was performed to identify latent classes of depression. Binary logistic regression analysis was employed to examine the association between the identified classes and related factors.

**Result:**

This study identified two distinct classes: the severe depression group (Class1, *N* = 162, 69%) and the mild depression group (Class2, *N* = 72, 31%). Binary logistic regression result demonstrated that, compared with the mild depression group, factors associated with the severe depression group included pain, fatigue, sleep quality, reluctance to distress disclosure and perceived low relationship quality.

**Conclusions:**

The current study provides evidence of the heterogeneity of depression among Chinese patients with NSCLC after surgery. Healthcare providers can develop tailored interventions by identifying the unique characteristics of each depression class. For patients in the severe depression group, interventions should focus on symptom alleviation, encouraging self-disclosure, and enhancing the quality of family relationships to improve their depression. As this study is cross-sectional, future research should validate these depression classes in larger and more diverse populations, both in China and globally.

**Trial registration:**

Chinese Clinical Trial Registry (ChiCTR, www.chictr.org.cn; registration no.: ChiCTR2400081943).

## 1. Introduction

According to estimates from GLOBOCAN in 2020, lung cancer ranks as a leading cause of 2.2 million new cancer cases and 1.8 million deaths globally [1]. The International Agency for Research on Cancer (IARC) predicts an estimated 28 million cases by 2040 [2]. In China, lung cancer is the leading cause of cancer death, with age-standardized mortality rates of 30.20 deaths per 100,000 people [3]. Non-small cell lung cancer (NSCLC) accounts for approximately 85% of all lung cancer cases [4]. Surgical resection is the cornerstone of treatment for NSCLC[5], significantly prolonging patient survival [6]. However, both surgical treatment and disease itself impose significant psychological burdens, such as depression, which can adversely affect patient outcomes [7,8]. Previous study indicated that the prevalence of preoperative depression in NSCLC patients is 12%, which increases to 61.89% postoperatively, suggesting a significant worsening of depressive symptoms following surgery [9,10]. Unresolved depression is associated with poorer treatment adherence, longer hospital stays, worse quality of life, poorer survival outcomes, and increased mortality [11,12]. Hence, early identification of depression may contribute to treatment decision-making and have significant public health and clinical implications [11,13].

Given that depression adversely affects the mental and physical health of patients with NSCLC after surgery, it is important to examine potential factors associated with depression. Previous studies have identified several factors that are strongly associated with depression in patients with lung cancer, such as gender, marital status, education level, and physical symptoms (e.g., pain, fatigue, and sleep quality) [14–16]. Moreover, the diagnosis of cancer not only impacts patients but also affects individuals within their social networks [15]. The Theory of Dyadic Illness Management points out that similar appraisals of symptoms by patients and caregivers lead to more active participation in disease management, resulting in better outcomes for both partner. Relationship quality and communication within dyads are key factors in the consistency of disease appraisal [17]. Social and familial relationships have emerged as significant factors influencing depression [18,19]. Self-disclosure, which involves sharing one’s own thoughts, emotions, and feelings with others [20], was associated with depression [21].

Previous studies have predominantly used total scores to evaluate depression in patients with NSCLC after surgery [12,14]. However, this approach has notable limitations, as it may overlook significant variations in specific symptom domains, including emotional, cognitive, and somatic manifestations, each of which may hold unique clinical significance. Due to the heterogeneity of individual experiences and characteristics, some patients may score higher on particular symptom dimensions despite exhibiting an overall lower severity level [22]. In the context of increasing emphasis on personalized care, there is a growing need for interventions tailored to specific patient profiles [23]. Latent class analysis (LCA), a person-centered methodological approach, offers a way to classify individuals into subgroups based on shared characteristics, ensuring homogeneity within groups and heterogeneity between them [24]. Despite its potential, the application of LCA in assessing postoperative depression among patients with NSCLC remains underexplored. This study aims to bridge this gap by employing LCA to identify distinct depression classes in patients with NSCLC after surgery and to investigate the sociodemographic, clinical, and psychosocial factors associated with these subgroups.

On these premises, the purpose of this study was to identify latent classes of depression in patients with NSCLC after surgery using LCA. In addition, we sought to explore the factors that influence these latent classes of depression, including demographic and clinical characteristics, physical symptoms, distress disclosure, and relationship quality. We hypothesized that there is heterogeneity in the experience of depression in patients with NSCLC after surgery and that specific factors are associated with each subgroup of depression.

## 2. Methods

### 2.1. Study design and sample

The study sample was drawn from the participants of the “The role of individual, dyadic and social factors in optimizing dyadic mental health after lung cancer surgery” recruitment survey. From February to April 2024, we conducted a cross-sectional study using a convenience sampling method. Patients with NSCLC who underwent thoracoscopic lobectomy and lymph node dissection were recruited from a tertiary hospital in Weifang, China. Data were collected from patients on the day of surgery until the third postoperative day. These patients had cancer stages ranging from Ia to IIa, with lobectomy as the primary surgical approach and segmentectomy or wedge resection performed in a minority of cases. The eligibility criteria for participants were as follows: (1) aged 18 or above; (2) without communication barriers and cognitive impairment. Participants were excluded if they (1) were diagnosed with a severe psychiatric disorder; (2) had extrapulmonary metastatic carcinoma or other comorbid diseases; (3) experienced a pneumonectomy.

### 2.2. Procedure

Participants completed pen-and-paper surveys simultaneously but in separate quiet rooms during their hospital stay. For participants who had difficulty reading the survey, researchers provided assistance in completing the questionnaires. Patients’ medical information was collected through chart reviews. The entire survey process took approximately 15 minutes.

### 2.3. Ethical considerations

This study adhered to the ethical standards for medical research involving human subjects, as outlined in the Declaration of Helsinki, and was approved by the Medical Research Ethics Committee of Weifang People’s Hospital (approval no: KYLL20240301−2). Written informed consent was obtained from all participants prior to their inclusion in the study. Additionally, the study was registered with the Chinese Clinical Trial Registry (registration no: ChiCTR2400081943).

### 2.4. Study variables

#### 2.4.1. Demographic and clinical characteristics.

Participants’ demographic data included age, gender, education level, employment status, marital status, and monthly income. Clinical characteristics included the number of complications.

#### 2.4.2. Depressive symptoms.

Depression was measured using the 9-item Patient Health Questionnaire (PHQ-9) [25]. This scale consists of 9 items, each scored from 0 (not at all) to 3 (nearly every day), with a total score ranging from 0 to 27. Higher scores indicate more severe depression. Total score of 0–4, 5–9, 10–14, and ≥15 correspond to no depression, mild depression, moderate depression, and severe depression, respectively.

#### 2.4.3. Patient symptoms.

Pain was evaluated using the Brief Pain Inventory (BPI) [26], The BPI includes three subscales: pain severity, affective interference, and physical interference. Each item is scored from 0 (no pain) to 10 (pain as bad as the patient can imagine). Average score of 0, 1–4, 5–6, and 7–10 indicate no, mild, moderate, and severe pain, respectively.

Sleep quality was assessed using the 8-item Athens Insomnia Scale (AIS) [27]. Each item is rated on a 4-point scale from 0 (none) to 3 (severe), with total scores ranging from 0 to 24. Higher scores indicate poorer sleep quality, with a score of ≥6 indicating insomnia symptoms.

Fatigue was measured with the Functional Assessment of Chronic Illness Therapy-Fatigue (FACIT-F) [28], a unidimensional scale consisting of 13 items. Each item is scored from 0 (not at all) to 4 (very much). Total scores ranges from 0 to 52, with higher scores indicating lower levels of fatigue.

#### 2.4.4. Distress disclosure.

The tendency to conceal or reveal psychological distress was measured using the Distress Disclosure Index (DDI) [29]. This scale includes 12 items rated on a 5-point Likert scale, ranging from 1 (strongly disagree) to 5 (strongly agree). Total scores range from 12 to 60, with higher scores indicating a greater willingness to disclose distress.

#### 2.4.6. Relationship quality.

Participants’ perceived relationship quality was assessed using a 15-item mutuality scale [30]. Each item is rated on a 5-point scale from 0 (not at all) to 4 (a great deal), with higher scores indicating better perceived relationship quality.

### 2.5. Data analysis

Data were analyzed using SPSS version 26.0 and Mplus version 7.4. Continuous variables with normal distributions were expressed as mean ± standard deviation (‾*x* ± *s*), while categorical variables were presented as frequency and percentage. LCA was employed to classify patients based on their depression profiles. Each item of the PHQ-9 scale, scored from 0 to 3, was dichotomized for LCA: scores <1 were classified as no depressive symptoms (0), and scores ≥1 were classified as the presence of depressive symptoms. To determine the optimal LCA model, several fit indices were considered: ①Akaike Information Criterion (AIC), Bayesian Information Criterion (BIC), and Adjusted Bayesian Information Criterion (aBIC). These criteria balance model accuracy and overfitting prevention, with lower values indicate better fit [31,32]. ②The entropy index, which quantifies class separability, ranges from 0 to 1, with higher values indicating greater accuracy; ③The fit criteria fail to provide statistical tests for demonstrating the superiority of one model over another. The Lo-Mendell-Rubin Likelihood Ratio Test (LMR) and the Bootstrap Likelihood Ratio Test (BLRT) are employed to compare a k-class model against a k-1 class model, providing robust determination of optimal class numbers in latent class analysis [33,34]. When *P*-values were significant (*P* < 0.05), it indicated that the k-class model was superior to the k-1 class model. In cases of inconsistency among these indices, the final decision on the number of classes was guided by practical implications and the requirement that each class contain at least 50 samples to ensure model accuracy [35]. Conditional probability plots were generated using GraphPad Prism software. Binary logistic regression analysis was conducted to evaluate the impact of each factor on the latent classes of depression, with results reported as odds ratios (ORs) and 95% confidence intervals (CIs). A nomogram was constructed using the “rms” package in R software version 4.4.0, based on significant factors identified in the regression analysis, to assess their association with depression. The nomogram’s discriminative ability was evaluated using the concordance index (C-index), where values range from 0.5 (no discrimination) to 1.0 (perfect discrimination). A significance level of *P* < 0.05 was applied throughout the analysis.

## 3. Results

### 3.1. Participant characteristics

A total of 234 patients with NSCLC after surgery participated in this study, with a response rate of 98.3%. Three questionnaires were excluded due to missing or incomplete data. The mean age of participants was 58.89 ± 10.27 years, with 38% (n = 90) male and 62% (n = 144) female (Table 1). Regarding education, 33% (n = 78) completed primary school, 31% (n = 72) completed junior high school, 28% (n = 65) completed senior high school, and 8% (n = 19) had a college degree or higher. Most participants were married (98%). The average monthly household income distribution was as follows: 19% (n = 45) earned ≤1000 RMB, 24% (n = 57) earned 1000–2999 RMB, 33% (n = 78) earned 3000–5999 RMB, and 23% (n = 54) earned ≥6000 RMB. Regarding postoperative complications, 76% (n = 179) had fewer than one complication, while 24% (n = 55) experienced one or more complications, including pneumonia, atelectasis, arrhythmia, hemorrhage, and chylothorax. 

**Table 1:** Sample Characteristics (N = 234).

Variables	%(*n*/*N*)
**Age (years)**	
≤45	10.26%(24/234)
46 ~ 60	46.58%(109/234)
>60	43.16%(101/234)
**Gender**	
male	38.46%(90/234)
female	61.54%(144/234)
**Marital status**	
Single	0.85%(2/234)
Married	97.86%(229/234)
Divorced	0.44%(1/234)
Widowed	0.85%(2/234)
**Education level**	
Primary school	33.33%(78/234)
Junior high school	30.77%(72/234)
Senior high school	27.78%(65/234)
College school	8.12%(19/234)
**Employment status**	
Employed	40.60%(95/234)
Retired	23.93%(56/234)
Unemployed	35.47%(83/234)
**Average monthly household income** (Chinese Yuan, RMB)	
≤1000	19.23%(45/234)
1000 ~ 2999	24.36%(57/234)
3000 ~ 5999	33.33%(78/234)
≥6000	23.08%(54/234)
**Number of complications**	
<1	76.50%(179/234)
≥1	23.50%(55/234)

### 3.2. Latent class analysis of depression

After evaluating four latent class analysis (LCA) models, we summarized their statistical fit metrics in Table 2. Based on a comprehensive assessment of the fit indices, the two-class model was identified optimal. As the number of classes increased, the values for AIC, BIC, and aBIC decreased (Table 2). However, in the three-class model, one class contained fewer than 50 patients, which could compromise accuracy. Therefore, the two-class model was selected as superior. This model also achieved an entropy value exceeding 0.8, and both LMR and BLRT yielded statistically significant results (*P* < 0.01).

**Table 2:** Latent class model fit indicators for depression.

Model (k-Class)	Log(L)	AIC	BIC	aBIC	Entropy	LMR	BLRT	Category probability
1-Class	−1074.29	2166.58	2197.67	2169.15	__	__	__	1
2-Class	−821.39	1680.79	1746.44	1686.22	0.91	<0.01	<0.01	0.69/0.31
3-Class	−776.89	1611.78	1711.98	1620.06	0.89	<0.01	<0.01	0.11/0.31/0.59
4-Class	−760.30	1598.61	1733.36	1609.75	0.90	0.02	<0.01	0.62/0.09/0.11/0.18

**Fig 1:**
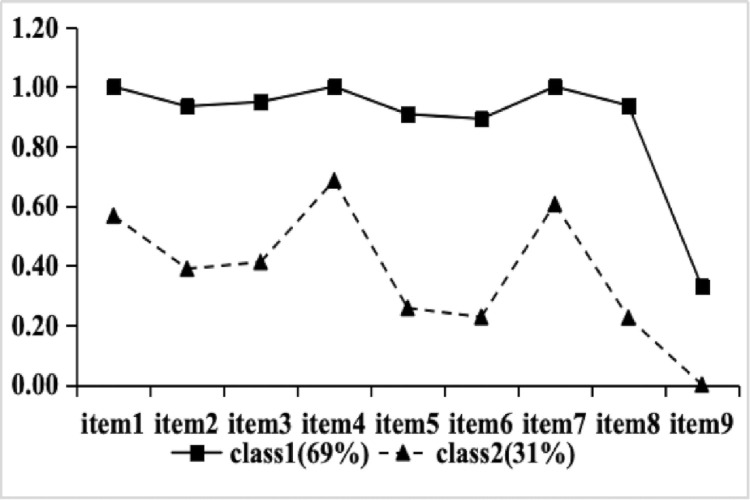
Response probabilities for two latent classes on each item of depression. *Note*: Item1: Anhedonia; Item2: Sadness; Item3 Sleep; Item4 Fatigue; Item5 Appetite; Item6 Worthlessness; Item7 Concentration; Item8 Movement; Item9 suicidal ideation.

The response probability graphs for the two latent classes across the nine depressive symptom items are presented in Fig 1. Based on the conditional probabilities for each depression, the two classes were named as follows: Class1, with a high conditional probability (scores > 0.5) for each item, was designated as “severe depression group”. This group indicates a pervasive and severe depressive state. Class2, which exhibited lower probabilities across most items but slightly higher probabilities for item 1 (Anhedonia), item 4 (Fatigue), and item 7 (Concentration), was named “mild depression group”. This pattern indicates that anhedonia, fatigue, and concentration difficulties represent the core symptoms of depression in this group, reflecting multifaceted distress at the physical, psychological, and cognitive levels.”

### 3.3. Comparisons between subgroups

#### 3.3.1. Analysis of different characteristics of patients with NSCLC after surgery in two latent classes of depression.

The two latent classes of depression in patients with NSCLC after surgery exhibited significant differences in the number of complications, pain, sleep quality, fatigue, distress disclosure, and relationship quality scores (all *P* < 0.05), as detailed in Table 3.

**Table 3:** Analysis of different characteristics of patients with NSCLC after surgery in 2 latent classes of depression.

Variables	Class1(*n* = 162), % (*n*/*N*)	Class2(*n* = 72), %(*n*/*N*)	*χ*^2^/*FET/t*	*P*
Age (years)			0.14^a^	0.93
≤45	9.88%(16/162)	11.11%(8/72)		
46 ~ 60	46.30%(75/162)	47.22%(34/72)		
>60	43.83%(71/162)	41.67%(30/72)		
Gender			1.87^a^	0.17
male	41.35%(67/162)	31.94%(23/72)		
female	58.65%(95/162)	68.06%(49/72)		
**Marital status**			1.89^b^	0.65
Single	0.62%(1/162)	1.39%(1/72)		
Married	98.15%(159/162)	97.22%(70/72)		
Divorced	0.62%(1/162)	0(0/72)		
Widowed	0.62%(1/162)	1.39%(1/72)		
**Education level**			0.51^a^	0.92
Primary school	33.95%(55/162)	31.94%(23/72)		
Junior high school	30.25%(49/162)	31.94%(23/72)		
Senior high school	28.40%(46/162)	26.39%(19/72)		
College school	7.41%(12/162)	9.72%(7/72)		
**Employment status**			3.66^a^	0.16
Employed	44.44%(72/162)	31.94%(23/72)		
Retired	23.46%(38/162)	25.00%(18/72)		
Unemployed	32.10%(52/162)	43.06%(31/72)		
**Average monthly household income** (Chinese Yuan, RMB)			2.27^a^	0.52
≤1000	16.67%(27/162)	25.00%(18/72)		
1000 ~ 2999	25.31%(41/162)	22.22%(16/72)		
3000 ~ 5999	34.57%(56/162)	30.56%(22/72)		
≥6000	23.46%(38/162)	22.22%(16/72)		
**Number of complications**			3.92^a^	0.05
<1	72.84%(118/162)	84.72%(61/72)		
≥1	27.16%(44/162)	15.28%(11/72)		
**Pain**	12.59 ± 2.96	9.61 ± 3.30	6.85^c^	<0.01
**Sleep quality**	10.78 ± 3.74	5.06 ± 2.98	11.47^c^	<0.01
**Fatigue**	24. ± 6.65	36.81 ± 4.89	−13.63^c^	<0.01
**Distress disclosure**	36.04 ± 2.599	39.63 ± 6.721	−5.99^c^	<0.01
**Relationship quality**	43.28 ± 6.898	47.19 ± 6.310	−4.38^c^	<0.01

a: χ^2^ test; b: *FET* = Fisher’s exact test; c: Independent Samples *t*-test.

#### 3.3.2. Multivariate analysis of two latent classes of depression in patients with NSCLC after surgery.

**Fig 2:**
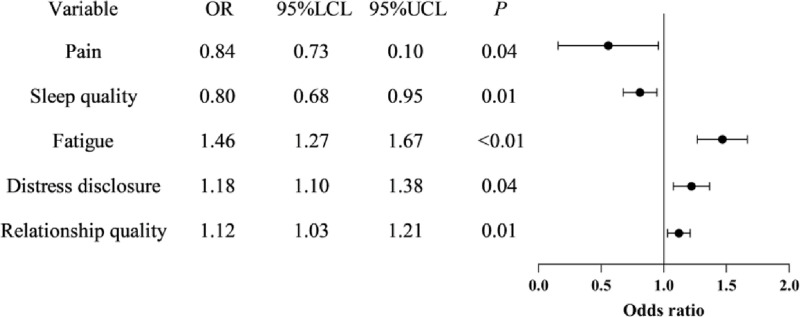
Odds Ratios Forest plot of binary logistic regression. Note: 95%LCL:95% Lower Confidence Limit: 95%UCL: 95% Upper Confidence Limit.

Binary Logistic regression analysis was conducted using the two latent classes as dependent variables and the statistically significant factors in univariate analysis as independent variables. The independent variables were coded as follows: Number of complications (<1 = 0, ≥ 1 = 1). The regression analysis results indicated that, compared to Class 1, patients with NSCLC after surgery who reported higher levels of distress disclosure and better relationship quality (odds ratios [ORs] = 1.01–1.38 and 1.03–1.21, respectively) were more likely to belong to Class1. Conversely, patients with higher pain levels, poorer sleep quality, and greater fatigue (ORs = 0.73–0.99, 0.68–0.95, and 1.26–1.67, respectively) were less likely to belong to Class2 (Table 4). Additionally, Fig 2 demonstrates that high levels of pain, poor sleep quality, and greater fatigue are risk factors for depression, while distress disclosure and relationship quality serve as protective factors.

**Table 4:** Multi-factor analysis of latent classes of depression.

	*B*	SE	Wald χ^2^	*P*	*OR*	95%CI
Constant	−20.92	4.65	20.21	<0.01	<0.01	——
Pain	−0.17	0.08	4.33	0.04	0.85	0.73 ~ 0.99
Sleep quality	−0.23	0.09	6.34	0.01	0.80	0.68 ~ 0.95
Fatigue	0.38	0.07	28.57	<0.01	1.46	1.27 ~ 1.67
Distress disclosure	0.17	0.08	4.21	0.04	1.18	1.01 ~ 1.38
Relationship quality	0.11	0.04	7.25	0.01	1.12	1.03 ~ 1.21
Number of complications	0.62	0.68	0.85	0.36	1.86	0.50 ~ 6.98

#### 3.3.3. Nomogram showing the association between significant factors and high-level depression in patients with NSCLC after surgery.

**Fig 3:**
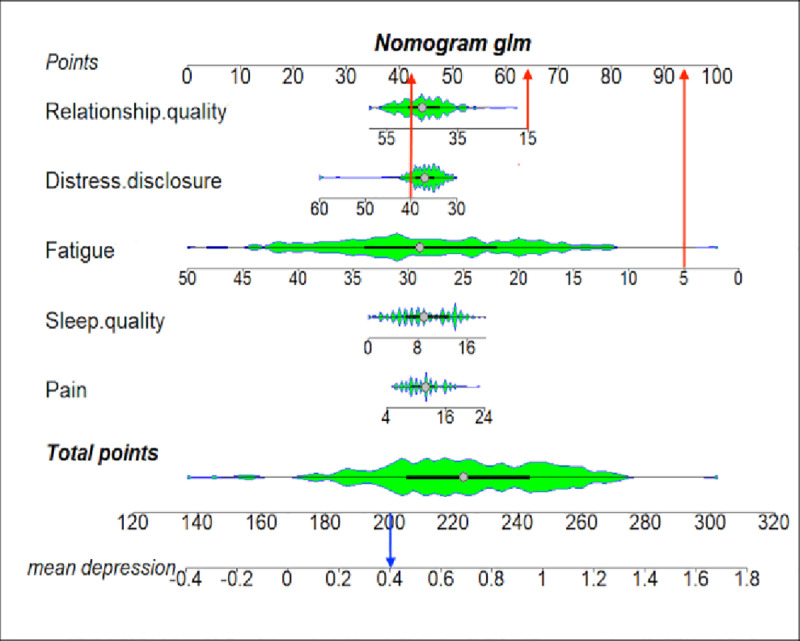
Nomogram for assessing the risk of depression in patients with NSCLC after surgery. Note: The nomogram was developed using five significant factors identified by binary logistic regression analysis. For each patient, a total score can be calculated by summing the individual scores of each factor, which corresponds to the risk of severe depression.

Based on binary logistic regression analysis, five significant factors—pain, fatigue, sleep quality, distress disclosure, and relationship quality (all *P* < 0.05)—were identified and incorporated into a nomogram (Fig 3) to visually illustrate their association with the risk of depression in patients with NSCLC after surgery. The length of each variable’s line represents its relative contribution to depression risk. The total score, calculated by summing the individual scores of all factors, corresponds to the risk of severe depression. The red and blue lines show an example, a fatigue score of 5 corresponds to 93 points, a relationship quality score of 15 corresponds to 64 points, and a distress disclosure score of 40 corresponds to 43 points, yielding a total score of 200. This total score corresponds to an association strength of 0.40 with the severe depression group. Among the variables, fatigue showed the strongest contribution, reflecting its significant impact on depression in patients with NSCLC after surgery. The nomogram demonstrates excellent discriminative ability (C-index = 0.95), effectively differentiating between patients with and without severe depression.

## 4. Discussion

Unlike previous studies that treated patients with NSCLC after surgery as a single homogeneous group, this study is the first to identify latent classes of depression and explore their associated factors using a person-centered approach. Two distinct classes were identified: a severe depression group (Class1) and a mild depression group (Class2), with Class 1 being the more prevalent. Class1, comprising 69% of participants, was characterized by a high probability of scoring across all nine depressive symptoms but slightly lower suicidal ideation. Surgical trauma and perioperative stressors often lead to significant psychological changes [36], underscoring the profound immediate impact of surgery on mental health. This finding aligns with a prior study reporting elevated rates of postoperative depression in similar populations, such as a study identifying a 61.89% depression rate in patients with NSCLC [10]. This may be linked to the assessment timing within 1–3 days after surgery. During this early recovery phase, factors such as physical pain, anesthesia effects, surgical trauma, and cancer-related concerns likely exacerbate depressive symptoms, compounded by physical discomfort and restricted mobility. By discharge, improvements in pain, mobility, disease understanding, and psychological adaptation may contribute to reduced depression levels. These findings suggest that preoperative exercise interventions could be a promising strategy to mitigate postoperative depression, as physical activity may improve psychological states through neurobiological and behavioral mechanisms [37]. Although Class2 represents a smaller proportion (31%), it remains clinically significant. This class exhibits symptoms such as anhedonia, fatigue, and concentration difficulties, reflecting challenges in psychological adaptation, physical functioning, and cognitive performance. Targeted interventions are needed to address these specific symptoms. For example, Integrative Body-Mind Training (IBMT), a traditional Chinese medicine-based intervention combining physical relaxation, breath adjustment, mental imagery, and mindfulness training, has been shown to alleviate fatigue and attention-related issues, offering a potential therapeutic approach for this population [38]. These findings highlight the importance of recognizing distinct depression profiles in patients with NSCLC after surgery, as such differentiation is essential for developing and implementing targeted interventions.

### 4.1. Latent classes and physical symptoms

The present study found that Class1 patients had significantly higher levels of pain, fatigue, and sleep quality problems. Edwards KA et al. [39] confirmed that these symptom are strongly associated with an increased risk of depression. Several potential mechanisms may explain this association. First, pain, fatigue, depression, and sleep frequently co-occur as a symptom cluster [40–42], which has been consistently associated with poorer patient outcomes [43]. The pathophysiology of these symptom clusters may involve shared inflammatory pathways, particularly the activation of pro-inflammatory cytokines such as IL-1α, IL-1β, IL-4, IL-6, IL-8, and TNF-α [44–49]. However, inconsistencies across studies underscore the need to consider factors such as cancer type, disease stage, treatment modalities, and assessment methods [47]. Further research is needed to explore the common mechanisms underlying these symptom clusters in patients with NSCLC after surgery, as targeting shared inflammatory pathways may offer therapeutic potential. Recent advances in symptom cluster research have incorporated network analysis, a novel paradigm enabling visualization and quantification of symptom interrelationships [50]. For instance, a network analysis study in patients with head and neck cancer identified depression and fatigue as core symptoms within psychoneurological clusters [51], aligning with our nomogram findings (Fig. 3) that highlight fatigue’s central role in depression. This is particularly relevant given fatigue’s predictive value for subsequent depression, insomnia, and pain in non-metastatic patients with cancer [52]. These findings underscore the clinical important of fatigue management in postoperative NSCLC care. Future research should focus on identifying core symptoms within postoperative symptom clusters in patients with NSCLC, enabling more precise interventions through targeted management of core symptoms. Additionally, the adverse effects of postoperative analgesics, such as opioids, can disrupt sleep patterns by reducing slow-wave and rapid eye movement sleep, leading to increased fatigue and poorer sleep quality [53]. This sleep deprivation may lower the pain threshold, further exacerbating depressive symptoms.

### 4.2. Latent classes and distress disclosure

The study reveals that those with lower levels of distress disclosure were more likely to be classified into Class1. Specifically, patients capable of expressing their concerns, emotions, and requirements regarding cancer-related matters reported lower levels of depression. This finding aligns with studies employing the Actor-Partner Interdependence Model, which indicate that self-disclosure by patients with cancer and their spouses is associated with reduced depression in both individuals [21]. A potential mechanism is that distress disclosure facilitates emotional catharsis, enabling patients to release negative emotions while gaining understanding and support from their spouses. This process strengthens emotional connections, alleviates loneliness and helplessness, and ultimately reduces depression. Moreover, the Systemic-Transactional Model highlights that stressors directly and indirectly impact both patients with cancer and their caregivers due to their interdependence, reinforcing cancer as a “we-disease” within families [54]. Sharing one’s worries and needs allows couples to more effectively navigate the experience together, reinforcing the concept of cancer as a “we-disease.” In addition, patients’ self-disclosure has been linked to positive outcomes in both physician-patient and therapist-patient contexts. For instant, when self-disclosure focuses on the patient’s needs and preferences—such as cultural or therapeutic considerations—it tends to yield more favorable outcomes compared to disclosure centered on the preferences of healthcare providers [55]. This is because self-disclosure allows patients to access disease-related medical information, promoting information exchange, practical planning, and adaption [56]. Moreover, lung cancer suffers from stigma due to its predominant association with smoking [57]. While treatment targets symptoms, stigma remains overlooked, causing discrimination, shame, and self-blame that lead to detrimental concealment and hindered support access. Facilitating safe self-disclosure enhances recovery by boosting hope and self-esteem while reducing shame [58]. Therefore, implementing distress disclosure interventions in clinical settings may effectively address stigma-related mental health challenges.

### 4.3. Latent classes and relationship quality

Patients with NSCLC after surgery experiencing lower relationship quality are more likely to be categorized in Class1. This finding aligns with existing evidence suggesting that patients with cancer perceiving lower relationship satisfaction are associated with reduced levels of depression [59]. Moreover, a study investigating relationship health in cancer dyads demonstrated that one partner’s perception of relationship quality significantly influences the other’s inflammatory markers (e.g., C-reactive protein [CRP]) and insulin resistance [60]. Higher relationship quality may attenuate adverse health outcomes, including inflammation and insulin resistance, potentially through oxytocin-mediated mechanisms and stress reduction. Building upon previous discussions, cancer represents a shared health challenge that extends beyond individual patients, exerting substantial impacts on both caregivers and dyadic relationships [61–63]. Previous studies have shown that patients who perceive higher relationship quality with their spousal caregivers experience significantly fewer depressive symptoms [64]. The reason for this may be attributed to the fact that greater marital satisfaction enables couples to develop healthier dyadic coping strategies, which are associated with lower levels of depressive symptoms. Furthermore, marital satisfaction, a key indicator of spousal relationship quality, plays a significant moderating role in the association between disease and depressive symptoms in couples. Higher levels of marital satisfaction can mitigate the impact of chronic disease on depression [65]. The Developmental-Contextual Model indicates that positive marital relationships are associated with more effective forms of dyadic coping (both supportive and collaborative), which not only mitigate the impact of adverse coping but also enhance couples’ psychosocial adaptation and disease management outcomes [66]. The findings offer valuable insights for healthcare professionals by emphasizing the critical need to consider the significant impact of couple relationships on health outcomes.

### Strengths and limitations

This study has strengths, such as utilizing latent class analysis to identify heterogeneous classes of depression among patients with NSCLC after surgery; however, it also has several limitations. First, due to its cross-sectional design, causal inferences cannot be made, and the dynamic aspects of depression and physical symptoms in this patient population cannot be analyzed. Additionally, since all variables, including depression and its associated factors, were assessed concurrently, the nomogram can only demonstrate associations rather than establish predictive relationships. Second, the recruitment of all participants from a single hospital may limit the generalizability of the findings. Third, the study did not include clinical sample information, such as biological markers, nor did it explore the relationship between the identified classes and such markers. Finally, while symptoms within a symptom cluster may interact with one another, the effects of symptom clusters on depression were not investigated. Future research should adopt longitudinal designs to collect data at multiple time points, which would allow for the establishment of predictive models, and further explore the validity of these classes in larger samples, as well as the role of symptom clusters and inflammatory processes in different depression classes.

## 5. Conclusion

This study identified two latent classes of depression among post-surgical NSCLC patients: the severe depression group and the mild depression group. Patients in the severe depression group were more likely to report elevated levels of pain, fatigue, and sleep qulity, perceive lower relationship quality, and exhibit reduced tendencies toward self-disclosure. Therefore, it is imperative to emphasize the need for tailored interventions such as personalized symptom management programs, communication skills training, and relationship-building strategies to address the unique needs of patients in the severe depression group. Future research should utilize longitudinal studies to examine the temporal stability and developmental trajectories of these depression classes, as well as the causal relationships between depression and sociodemographic, clinical, and psychosocial factors. Additionally, multi-center studies are recommended to enhance the generalizability of findings and further validate the effectiveness of targeted interventions across diverse patient populations.
